# Establishing a clinical ethics support service: lessons from the first 18 months of a new Australian service – a case study

**DOI:** 10.1186/s12910-023-00942-9

**Published:** 2023-08-11

**Authors:** Elizabeth Hoon, Jessie Edwards, Gill Harvey, Jaklin Eliott, Tracy Merlin, Drew Carter, Stewart Moodie, Gerry O’Callaghan

**Affiliations:** 1 School of Public Health, The University of Adelaide, PO Box 5005, Adelaide, SA 5005 Australia; 2 Adelaide Medical School, The University of Adelaide, PO Box 5005, Adelaide, SA 5005 Australia; 3 College of Nursing and Health Sciences, Flinders University, PO Box 2100, Adelaide, SA 5001 Australia; 4 Central Adelaide Local Health Network, Adelaide, SA 5000 Australia

**Keywords:** Clinical ethics committees, CECs, Clinical ethics, Qualitative approach

## Abstract

**Background:**

Although the importance of clinical ethics in contemporary clinical environments is established, development of formal clinical ethics services in the Australia health system has, to date, been ad hoc. This study was designed to systematically follow and reflect upon the first 18 months of activity by a newly established service, to examine key barriers and facilitators to establishing a new service in an Australian hospital setting.

**Methods: how the study was performed and statistical tests used:**

A qualitative case study approach was utilised. The study gathered and analysed data using observations of service committee meetings, document analysis of agendas and minutes, and semi-structured interviews with committee members to generate semantic themes. By interpreting the thematic findings in reference to national capacity building resources, this study also aimed to provide practice-based reflections for other health agencies.

**Results: the main findings:**

An overarching theme identified in the data was a strong commitment to supporting clinicians facing difficult patient care decisions and navigating difficult discussions with patients and families. Another key theme was the role of the new clinical ethics support service in providing clinicians with a pathway to raise system-wide issues with the organisation Executive. While there was strong clinical engagement, consumer and community participation remained a challenge, as did unresolved governance issues and a need for clearer policy relationship between the service and the organisation.

**Conclusions: brief summary and potential implications:**

Considering these themes in relation to the national capacity building resources, the study identifies three areas likely to require ongoing development and negotiation. These are: the role of the clinical ethics support service as a link between the workforce and the Executive; the incorporation of consumers and patients; and ethical reasoning. To improve the effectiveness of the service, it is necessary to increase clarity on the service’s role at the governance and policy level, as well as develop strategies for engaging consumers, patients and families. Finally, the capacity of the service to reflect on complex cases may be enhanced through explicit discussions of various different ethical frameworks and ways of deliberating.

**Supplementary Information:**

The online version contains supplementary material available at 10.1186/s12910-023-00942-9.

## Introduction

At a time when health systems are under great pressure, incorporating clinical ethics support into everyday medicine is critical to addressing contemporary community expectations about access to and delivery of health care, and supporting health professionals to deliver technologically and ethically complex medicine [1]. Although the importance of clinical ethics in a contemporary clinical environment is established, its practical application in Australia in the form of formal clinical ethics support services (CESS) has been ad hoc, with a limited number of established CESS in the health care system [2]. This lack of a coherent strategy to formalise CESS in health services is in contrast to northern America and many northern European countries, where such services are well established, in some cases for over twenty years [3–5]. In recognition of the need for a national approach, in 2015 the Australian Health Ethics Committee (AHEC) of the National Health and Medical Research Council (NHMRC) published a consensus statement [6] and resource manual designed to support more systematic provision of clinical ethics support in Australian health care organisations [1]. This capacity-building initiative complements the recent inclusion of CESS as a requirement of formal hospital accreditation guidelines [7].

In jurisdictions where CESS are well established, while there have been empirical studies, those that critically examine how these services work in practice are limited. Instead, descriptive studies about CESS prevalence, structure, composition and function have been prominent [8]. Where formal evaluation has been conducted, surveys using the domains of “satisfaction, ethicality (ethical acceptability), education and conflict resolution” have commonly been used [5]. Multi-centre US and national Norwegian survey-based studies of CESS have reported high levels of stakeholder satisfaction [9, 5]. While such study designs provide some measure of effectiveness and comparability between CESS, it is acknowledged that these data tend to provide ‘thin descriptions’ of the content discussed by CESS [8], and reveal little about how CESS operate in practice and the effectiveness of these practices and processes. Several recent studies have turned to qualitative approaches to gain deeper understandings of practices involved in CESS and to expand methodological approaches for assessing the quality of a CESS [5, 8, 9]. These qualitative studies have provided insights about the nuanced ways in which these services work for stakeholders, including differences in perspectives, and provided rich analyses of what quality in clinical ethics consultations and deliberations looks like [5, 8, 9]. In the recent study by Kana et al., [8] two domains of quality (satisfaction and value) were found to be important for health professionals who had used ethics consultation services, with qualitative analysis emphasising how the process of consultation created valuable moral space, promoting thoughtful and ethical responses to dilemmas in patient care [8]. The importance of stakeholder participation (i.e. patents, relatives and professionals), even when their presence brought tension and conflict to the consultation and possible dissatisfaction with outcomes, has also been highlighted as a key contributor to well-functioning CESS [5].

Building on these recent process-focussed studies of established CESS outside of Australia, this research was designed to systematically follow and reflect upon the first 18 months of activity by a newly established CESS, to examine the key barriers and facilitators to establishing a new service in an Australian hospital setting (at a time when the COVID-19 global pandemic emerged), and provide direction to the organisation on how to further develop and improve the service. By further interpreting the empirical findings in reference to the foundational NHMRC clinical ethics capacity building resources [1], this study also aimed to provide practice-based reflections for use by other health agencies that may be interested in developing their own CESS.

## Methods

### Study design and setting

To gain in-depth understandings of how a local clinical ethics service developed in a challenging and complex environment, a qualitative case study approach was utilised [10]. Case study designs are particularly useful to facilitate the exploration of complex phenomena within a natural setting through the use of multiple research methods [11]. Because the agency had specific questions about how this new service worked in practice, the research design was pragmatic, with the aim that the study’s findings contribute to the service’s development and decision-making processes. To this end, the study gathered and analysed data using observations, document analysis, and semi-structured interviews, to generate semantic themes [12] reflecting an understanding of how this CESS established itself within its local health network setting, and to identify the key enablers and challenges in developing the capacity of this new CESS. Data were gathered between May 2020 and December 2021 and were confined within a newly established service in a large Australian metropolitan local hospital network serving a population of almost 500,000. The CESS served this hospital and several other centralised regional tertiary and quaternary clinical services. This service was established in response to the emerging COVID-19 pandemic in anticipation that clinical ethics would be a key aspect to delivering ethically justifiable health services at this time. The NHMRC consensus statement and resource manual were used to guide the development of the new service, which took the form of a Clinical Ethics Committee (CEC) [1]. The committee’s membership included consumer advocates and representatives, a variety of senior clinicians, representatives from the organisation’s senior management including in-house legal counsel, First Nations representatives, university academics, non-health-care public servants, and a health ethicist. It is worth noting that in Australian health services research and health services delivery, ‘consumer’ is a widely-used umbrella term for patients, carers, and health services users, as well as potential consumers (i.e. the community and public), reflecting a shift away from a conception of health service users as passive patients. The research team included two partner investigators who were both CEC members. To avoid a conflict of interest, these partner investigators did not have access to primary data from interviews (neither audio nor transcripts), but were involved in analysis and interpretation once initial themes were generated.

### Recruitment

Given the bounded and small sample in this case study, participant recruitment included several layers of informed consent. First, permission was sought from all CEC members and occasional meeting attendees for a researcher to observe the committee meetings in real time. Potential participants were advised that where informed consent was not provided by all, the researcher would not observe the meeting but instead have access to the agendas, minutes and supporting documents (redacted for privacy) of the committee meeting, with consent of the chairperson. Because all members agreed to be observed, in most instances the ‘back-up’ plan of reviewing deidentified documents was not required. However, the CEC did invite occasional participants, and where consent for these participants could not be arranged ahead of time, the researcher withdrew from the meeting and instead received redacted minutes. Second, all committee members and staff who had engaged with the service were invited, via the secretariat to the committee, to participate in a semi-structured interview. It is noteworthy in terms of sample generation, that although clinical staff who had engaged with the service (through case review consultations or providing expert advice) were also invited by the secretariat to participate in the research, none followed up this invitation. Participant characteristics are not detailed because providing detail about discipline, age, gender, or seniority/years of practice could compromise participant anonymity.

### Patient and public involvement

There was no patient or public involvement in the design of the study. The service forming this case study included membership representation from consumers and community, and as such they were invited to participate in the study. The study findings were presented to the consumer and patient advocate reference group at the local health network and their feedback, including practice-focussed recommendations, are now being considered and actioned by the CEC.

### Data collection and analysis

Data collection began with a field observation phase, with a qualitative researcher (EH) observing the CEC meetings from May 2020 to December 2021, using Silverman’s approach to inform guiding questions: What are people doing? What are they trying to accomplish? How exactly do they do this? What assumptions do they make? [13] Observations of these online meetings were recorded on a template (see Supplementary table 1), which formed the basis of reflective field notes generated by the researcher and discussed by the team. These reflective notes were supplemented by a review of CEC meeting agendas, minutes, and supporting documents. Observations were not extended to case review consultations performed by the service as the researchers did not want to further burden patients or their families by asking for their consent to participate in the study. The CEC tried to minimise the research team’s exposure to patients’ data by only providing case consultation meeting notes, which were redacted to decrease identifiability. This meant that the researchers had some access to non-identifiable patient data as necessary to study the work of the service. Preliminary learnings and themes generated from reflexive thematic analysis [14] of data from the observational phase informed the development of questions for semi-structured interviews with CEC members, conducted between August and October 2021 by EH. The questions focussed on the experiences and expectations of the CEC members, the main achievements and challenges for the service, the processes of the committee (including deliberation and how the representation of views were managed in meetings), and its place within the organisation (see Supplementary table 2 for interview schedule). All 20 CEC members were contacted, and 12 agreed to be interviewed. Interviews were conducted in person or via telephone or video teleconference, according to participants’ preferences. All interviews were digitally audio-recorded and transcribed verbatim.

The analysis of interview data was informed by thematic analysis [14] and conducted using NVivo 12 software (QSR International Pty Ltd, Doncaster, Victoria, Australia). Interview transcripts were read and re-read to enable immersion in the data. Initially, semantic inductive coding [12] of two interviews was conducted by two researchers (EH and JEd), who worked collaboratively to develop thinking about codes and patterns within the data. Coding of all interview data was then undertaken. Preliminary generation of themes was conducted through reflective discussion between two researchers (EH and JEd), to challenge thinking, build depth to the themes and enhance rigour. Discussions about emerging themes then occurred with all researchers, including the partner investigators, until consensus about the themes was reached. The findings were presented back to the CEC to encourage their reflections on whether the themes resonated with the collective experience of the service, with some subsequent refinement of the theme labels and descriptions.

## Findings

**Fig. 1:**
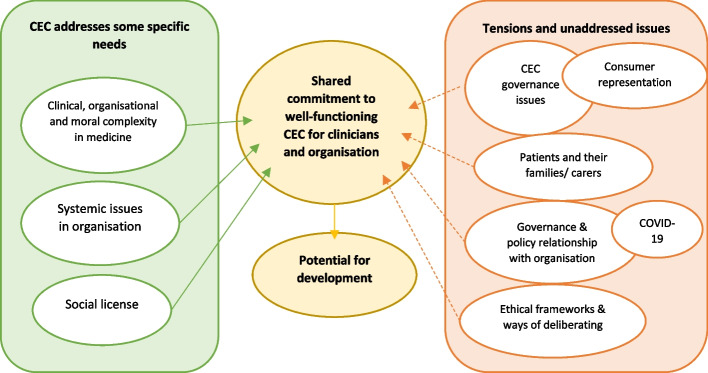
Final key themes generated by analysis, including relationships between themes

Observations of committee meetings occurred from June 2020 through to December 2021 (9 meetings). Document analysis of meeting agendas and minutes for this same time period was undertaken, and semi-structured interviews with 12 participants were conducted between August and September 2021. The overall thematic map of findings from these data collection phases is shown in Fig. 1.

An important overarching theme identified was the *strong commitment to supporting clinicians,* and a key contributing factor to this was a desire by many in the service to *support clinicians who face clinical and moral complexities in their work*.

[W]e've offered ways that the team could be supported in dealing with these complex cases, because they are complex cases, you can see how relieved they are and how they feel supported by the organisation. (Participant 1)

There was general agreement that a key role of the CEC is to support clinicians with ‘tricky’ and ‘difficult’ clinical decisions about patient care, with observations such as:

Supporting teams when they're faced with the cases that haunt you. […] I thought it was better that we influence tricky decisions than the poor people at the coalface … the RMO [resident medical officer] or registrar at two o’clock in the morning deciding who got the last ventilator. (Participant 4)

Several participants noted that their decision to nominate for the new service was driven by a desire to support their peers through the COVID-19 pandemic. Another contributing factor to this overall theme was the role of the service in *highlighting systemic issues* within the organisation. It was widely recognised that the CEC provided a new forum and pathway to raise with senior managers system-wide issues that could be usefully clarified with an ethical lens:

[Clinicians are] starting to use us a little bit more as a resource for the really complex cases, and also as a way of highlighting some of the system issues that don't have a pathway to management particularly well at the moment. (Participant 8)

and

It’s really important that even if you resolve the issue, we still talk about these because then it’s documented …it’s flagged as a recurring concern, and this is something the organisation needs to work to address. So it is a mechanism to raise things that need to be addressed. (Participant 8)

A further contributing factor identified within this overall theme of *a strong commitment to supporting clinicians* was *supporting clinicians in negotiating care with patients and their families* in the immediacy of managing clinical care:

I mean, an ethical issue arises usually because there is a disagreement, whether it's between patients, patient’s families, different treating teams, anyone who's sort of a stakeholder in the situation, as soon as there's a difference of opinion, that's where the Ethics Committee can play a role. (Participant 10)

Relationships between clinicians and patients and their families are subject to the expectations of consumers and their families, and in the contemporary health setting, where community members are encouraged and often empowered to advocate for themselves, it was understood that clinicians may need organisational support in these negotiations.

[When] … it’s like, “Well, I don't trust you. You gave me the wrong advice and you’re not listening to me. I want this and you’re not letting me have it.” Whatever it is, then that’s really difficult for clinicians. (Participant 7)

Many CEC members expressed the desire to ‘be there’ for their colleagues and acknowledged that because the service sits within an established formalised governance system, there is the potential to respond to their colleagues’ needs at an organisational level. As such, there was a strong shared commitment to developing a well-functioning forum, linking the clinical ethics needs of staff to senior management.

I think it’s very credible in terms of the breadth of clinical expertise as well as Executive buy-in (Participant 9)

and

[A]fter we've had the session with them and we've discussed and we've offered ways that the team could be supported in dealing with these complex cases, because they are complex cases, you can see how relieved they are and how they feel supported by the organisation. So I think the Committee provides an opportunity for the organisation to sort of get together and support the clinicians and show that we are an organisation that does get behind our people. (Participant 12)

Although there was strong shared commitment to a well-functioning service, three further themes related to tensions and unaddressed issues that require attention to extend the clinical ethics capacity of the CEC and the organisation. The first related to *an unresolved governance and policy relationship* between the CEC and the organisation. This relationship was recognised in the writing of the initial Terms of Reference, but the role of the CEC in policy was not explicitly explored in later discussion.

I think I'd be worried that if I had … it as kind of a big discussion about whether [administrative or policy matters are] a core focus of the Committee, that there might be some pushback from people who want it be focused only on direct clinical encounters. So I included that in [the Terms of Reference], which I sent around for people's opinions, but I think we never really sat down and had a discussion about, do we want that to be a key focus of the Committee. (Participant 2)

There were divergent views within the CEC about whether its role should be to influence or contribute to the wider policy agenda of the organisation, especially considering established procedures for ethical consideration within the organisation.

I don't know if ethics has a role in [policy]. I don't know how you engage that. So we have different committees in the hospital who look, for example, at high-cost pieces of equipment and whether that gets purchased or not. So it may not be a role for this Committee. But I think, you know, I guess the impetus for the forming the formality of this Committee in the first place was around the distribution of resources around COVID. (Participant 4)

and

I firstly don’t think the role is of the Committee is to have a say. It would be to give advice or give an opinion when there was a complex issue [….] I personally don’t think the role of the Committee is to develop protocols and things like that, pathways. We are here to listen and give advice. Well I think that’s what the role is. On an ethical basis. (Participant 11)

Compared to other perspectives that advocated a stronger policy role:

I would have liked to have seen us – this might be a question coming up – but I would have liked to have seen us be more involved in review of more policy-type frameworks, because I think there are probably a lot of policies that get escalated, approved, without really any consideration as to whether this is fair, just, ethical, whatever words we use. (Participant 1)

To date, the main focus of the CEC has been case review, but beyond that direct and specific support for clinicians, for some members it was ‘still ambiguous as to what we’re supposed to be contributing to’ (Participant 1). This stemmed from a lack of clarity about broader questions of governance and purpose. Despite universal acknowledgement of strong Executive support, questions still to be resolved included: What role does the service have in policy development? What expectations does the organisation’s Executive have of the CEC? How can the outcomes of the case studies or minutes be used by the organisation beyond case review, to improve clinical ethics reasoning?

We’ve created our own … pathway or decided how we think, we as a Committee, we could influence or improve things. But I’d be interested in whether that aligned with the institution’s goals when we were created. (Participant 1)

A further key theme highlighted *ongoing uncertainty about how to effectively engage consumers.* Despite a widespread philosophical commitment to involving patients, their families, and the wider community (consumers and other stakeholder agencies), and strong consumer advocacy throughout CEC discussions and deliberations, there remained uncertainty about some of the practical application of this intent. For instance, while the role of the committee in supporting clinicians with difficult medicine was emphasised, there was less certainty about how they could support and empower even the consumers at the heart of a case. Similarly, the broadness of the term ‘consumer’ meant that varying interpretations and contextual usage amplified challenges in defining the roles and positions of consumers with the service. This theme connects closely to the *unresolved governance issues*, and raised the following questions: How can a wide range of community voices be best represented in the service? What are the mechanisms for consumers and their families to initiate a clinical case review, and how should consumers be represented at a case review meeting?

And I'm a really keen supporter of consumer voice, but …. Just getting a consumer for the sake of a consumer who’s not—doesn't have a background could be problematic for the individual as well as for the process … But the level of conversation and unpacking of individual cases may be challenging for a, sort of a, for want of a better word, a naive consumer. To just have a consumer rep on the Committee. (Participant 6)

Further, how should the service report advice that is generated by a clinical ethics case review? In what form and where should such advice be stored, taking account of the need to balance the need for transparency of committee processes, while ensuring confidentiality of consumer information?

There was a lot of debate about who would document that opinion [generated by the committee’s case review process], where would it sit, would it actually be available to the general patients still, and their families through sort of the rights to freedom of information? (Participant 1)

and

I just wonder whether [we should recruit] a citizen, who is not an advocate for patients and who’s not a clinician. (Participant 3)

Many of the CEC members acknowledged that translating the CEC’s intent to involve consumer and patient voices into practice was challenging and required careful development of appropriate policies and procedures by the CEC and the wider organisation.

A final theme related to a *need for explicit discussions of different ethical frameworks and ways of deliberating.* Although some members didn’t see the need for further theoretical discussion of ethical frameworks, others argued that in order to improve ethical deliberations, members should be explicit about the ethical approach being drawn upon in their reasoning, and there should be opportunities to discuss alternative ethical approaches.

I think it’s really important to think about different ethical approaches and that people can come to the same or different decisions and still be coming from an ethical perspective, but perhaps a different model of ethical reflection. (Participant 4)

To date, there has been limited explicit discussion about the different *substantive* ethical orientations that members may be bringing to deliberation (e.g. consequentialist or deontological positions), as well as the *procedural or deliberative* frameworks that could be employed in deliberation, and this was identified as a potential source of tension or misunderstanding between CEC members.

## Discussion

So, what might a prospective CESS expect to grapple with in its initial stages of development? The NHMRC Consensus Statement and Resource Manual (henceforth ‘Guidelines’) [1] were used as guides in the establishment and development of the CEC, and several of the key themes discussed above relate to elements of this resource that we recommend prospective CESS consider closely. In an established tertiary health setting, where case referrals will emerge from the predictable needs and capacities of the workforce and organisation, we consider the following three areas to be likely domains of negotiation and development: the CESS as link between workforce and Executive; incorporating consumers and patients; and ethical reasoning.

### The CESS as link between workforce and Executive

Having experienced difficult or complex medicine, and wanting to support their colleagues experiencing the same, presents an important motivation for those clinicians who nominate for CESS membership. However, members place value both in assisting colleagues with specific cases and in benefitting the organisation, by improving its ethical culture and highlighting systemic issues that may require Executive attention and resolution. Often, case consultation requires attention to this linking function: as the Guidelines note, a final question to ask in the consultative process is: ‘are there any individual, organisational, systemic, educational or policy issues to follow up as a result?’ (‘Tools for consultation and case analysis’, Sect. 4.2, p. 21). Our findings reinforce the relevance of this question.

The CEC is viewed by its members as a specific and independent forum in which the medical, nursing, and allied health workforce can seek the support of colleagues. By virtue of being integrated in the organisational structure, the service also provides the opportunity for the organisation to respond to unaddressed patient and workforce needs. A ‘well-functioning’ CESS, then, will have direct and strong links to and support from the Executive and will have considered closely where it fits in the governance structure. Where CESS members come together with a shared commitment to a well-functioning and useful service, without a clear sense of how their work links the day-to-day of ‘difficult medicine’ to governance and policy, member engagement may falter. Similarly, members who prefer practical clinical discussion with clear outcomes may experience frustration if operational and process questions are not resolved quickly. Section 3.1 of the Guidelines (‘Governance, accountability and reporting’, p. 13) provides a useful starting point for thinking through some operational issues in the form of specific process questions relating to, for example, the storage of case notes, engagement with the hospital community, and communication with Executive. Answers to these questions will flow from an understanding of the CESS as providing a crucial link between the day-to-day of ‘difficult medicine’ and more overarching concerns of governance and policy.

### Involving consumers

When developing a CESS, the question of how to incorporate consumer, community, and patient and family voices is critical. The Guidelines do not emphasise community representation as a necessary component of committee membership (‘Membership considerations’, Sect. 2.8, p.11), however, there is a strong trend towards incorporating consumer voices in health settings [15] and the membership of the service being studied also considered it a priority. Recognising the importance of membership from outside the health service workforce raises key questions about the nature and purpose of consumer representation, with answers potentially depending on the specific setting and goals of the CESS. What is the difference between a community voice, a consumer voice, and a patient voice, and do all of them need to be represented? If part of the purpose of the service is to support clinician-peers with difficult medicine, in what ways is support extended to consumers and families? How might a consumer, or their advocate, raise and/or be involved in their own case consultation? What are members’ expectations about what a consumer voice is or does in practice, whose voice it should be, and when and where it should be heard? These questions can be contentious, sensitive, and difficult to resolve, and may require periods of reflection and adjustment as a service matures.

### Ethical reasoning

The Guidelines (‘Tools for consultation and analysis’, Sect. 4.2, p.18) note that diverse formal approaches to ethics consultations share an aim to ‘widen the sources of moral input’, to foster reflection on one’s own and others’ moral views and biases, and to foster learning from shared experience. Due to the predominantly clinical nature of a CESS, one might expect that membership will be primarily clinical, with members who by training and practice are comfortable with principles-based, linear approaches to ethical dilemmas [16]. However, given the diversity of disciplines possibly represented in the service, as well as the presence of community, pastoral, and cultural representatives, case deliberation may involve discussion across substantive ethical positions (e.g., care or relational ethics, virtue ethics, or religious ethics). Similarly, different deliberative tools and processes may also be prioritised by some members. Being explicit about the features and orientations of different approaches to ethical reasoning may help avert any misunderstandings arising within the service. In addition, explicit discussions of alternative ethical frameworks and ways of deliberating may provide insights for services both in negotiating differing viewpoints and in incorporating ethical insights from a widened range of sources relevant to the healthcare setting. To foster such discussions, the Guidelines (‘Membership considerations', Sect. 2.8, p.11) recommend actively recruiting members with formal ethics training (e.g. clinical ethicists, academic ethicists), and exploring the full range of deliberative frameworks identified by the Guidelines (‘Tools for consultation and analysis’, Sect. 4.2, p.19). In this case study, while there was active recruitment of members with formal ethical training, there were significant constraints limiting the explicit discussion of ethical frameworks in the CEC. These included the COVID-19 pandemic as the original impetus for the service’s development, as well as the time restrictions of committee meetings and prioritisation of case studies as agenda items, and high demands on the time of CEC members. This made implementing the Guideline advice challenging. It could be expected that other new services might face similar challenges.

Finally, several limitations in the study design implementation should be acknowledged. Although the study aimed to include data from consumers and staff who had interacted with the service, the researchers were unsuccessful in recruiting participants from this stakeholder group. This limits the range of included experiences and perspectives to only the CEC members and it is acknowledged that a future priority for the CEC will be to evaluate the quality of this new service from a wider perspective. In relation to transferability of findings, it is acknowledged that, while this case study design allowed in-depth exploration of many processes operating in the specific context of this service in a particular health organisation, to ensure anonymity, contextual details about the organisational setting have been limited in the reporting of the study. This may affect the ease of transferring the findings to a different health context. In relation to understanding the impact of COVID-19 on this service’s development, it is important to acknowledge that as Australia closed its international borders for 2020–21, the impact of COVID-19 on the health system was different to many other countries, and although the system was severely stressed, it was not overwhelmed, and care rationing was not as much of a problem here as it was in some European countries or the US.

## Conclusion

The study has provided the opportunity to describe the challenges and enablers which accompany the establishment of a CESS in real time. Consumer and community participation remain a challenge in spite of strong clinical engagement. Our findings provide a pathway for subsequent deliberate development of CESS, particularly in terms of ensuring the relationship between the service and senior management is made explicit, and that ethical frameworks to guide and support ethical deliberation and advice formation are introduced. Further opportunities exist for developing resources to support consumer and community participation.

### Supplementary Information

**Additional file 1: Supplementary Table 1.** Observation template for committee meetings.

**Additional file 2: Supplementary Table 2.** Interview questions.

## Data Availability

The datasets generated and/or analysed during the current study are not publicly available due to the small number of participants in the study who may be at risk of being identified by the in-depth interview data. Summary results data are available from the corresponding author on reasonable request.

## Abbreviations

AHEC: Australian Health Ethics Committee
CEC: Clinical Ethics Committee
CESS: Clinical ethics support services
NHMRC: National Health and Medical Research Council
