# Supplementary material for: Establishing a clinical ethics support service: lessons from the first 18 months of a new Australian service – a case study
Source: BMC Med Ethics. 2023 Aug 11;24:62. doi: 10.1186/s12910-023-00942-9 (PMC10422737; doi:10.1186/s12910-023-00942-9)
Supplement: Supplementary file 1 — Additional file 1: Supplementary Table 1. Observation template for committee meetings. [file 12910_2023_942_MOESM1_ESM.pdf]

Supplementary Table 1: Observation template for committee meetings.

Committee Meeting Observational Template DATE____________________ Observer____________________________

Time and setting	Meeting activity	Behaviours	Interactions<br>nonverbal behaviours and verbal behaviours
