# Supplementary material for: Establishing a clinical ethics support service: lessons from the first 18 months of a new Australian service – a case study
Source: BMC Med Ethics. 2023 Aug 11;24:62. doi: 10.1186/s12910-023-00942-9 (PMC10422737; doi:10.1186/s12910-023-00942-9)
Supplement: Supplementary file 2 — Additional file 2: Supplementary Table 2. Interview questions. [file 12910_2023_942_MOESM2_ESM.pdf]

Supplementary Table 2: Interview questions.

Study Title: How does the CALHN Clinical Ethics Committee support decision making processes for clinicians and managers in a global pandemic – A case study.

Questions for semi structured interviews with committee members

To begin with, a few questions about your background

a) Your profession
b) Age
c) Do you have any previous experience of clinical ethics committees, if so what was this, and for how long?
d) Any training in moral philosophy or ethical reasoning? If so, what was this?

Moving on to your experiences of this CEC

1. How did you come to be part of this committee?

2. What were you hoping to bring to the committee?

3. Thinking back over the first year of this committee, from your perspective, what have been the main achievements for the committee?

4. And what have been the key challenges for the committee?

Moving on to some more specific questions

5. How do items for consideration come to the committee?
    a. Have you raised any items, how was that?

6. When considering items brought to the committee, what resources (skills, evidence, literature) have you used to support your deliberations?

7. How have you found the negotiation of differing opinions within the committee?

8. In terms of the decision-making process how have you found this?

Supplementary Table 2: Interview questions.

- Opportunities for discussion
- Opportunities for alternate view points
- Confidence to raise items

9. How have you found the composition of committee membership - have the right people been at the table – if not who missing?

10. Meetings have been almost exclusively online – how has this influenced the functioning of meetings and the committee?

11. And the observational research that I have conducted?

12. Do you think the CEC has a role in priority setting within a health system – what would that look like?

13. Is there training/ educational or other resources that might be of use to support your role and the overall committee's work? What would that look like?

14. Has the committee operated as you expected it would? Why is that?

OR (will see how goes in first interview)

Thinking about how the committee has worked over the its first year, if you had the opportunity to set up an equivalent committee somewhere else, what things would you want to see in place?

15. What should be the priorities for the next year, for the committee?
